# Nurse Competence on Physiologic Monitors Use: Toward Eliminating Alarm Fatigue in Intensive Care Units

**DOI:** 10.2174/1874431101711010001

**Published:** 2017-04-14

**Authors:** Azizeh K. Sowan, Ana G. Vera, Elma I. Fonseca, Charles C. Reed, Albert F. Tarriela, Andrea E. Berndt

**Affiliations:** 1 School of Nursing, University of Texas Health, San Antonio, 7703 Floyd Curl Dr. - MC 7975, TX 78229, USA; 2 University Health System, 4502 Medical Drive, San Antonio, Texas 78229, USA

**Keywords:** Nurse competence, Physiologic monitors, Alarm fatigue, Intensive care units, ICUs, Survey

## Abstract

**Background:**

Studies on nurse competence on alarm management are a few and tend to be focused on limited skills. In response to Phase II of implementing the National Patient Safety Goal on clinical alarm systems safety, this study assessed nurses’ perceived competence on physiologic monitors use in intensive care units (ICUs) and developed and validated a tool for this purpose.

**Methods:**

This descriptive study took place in a Magnet hospital in a Southwestern state of the U.S. A Nurse Competence on Philips Physiologic Monitors Use Survey was created and went through validation by 13 expert ICU nurses. The survey included 5 subscales with 59 rated items and two open-ended questions. Items on the first 4 subscales reflect most common tasks nurses perform using physiologic monitors. Items on the fifth subscale (advanced functions) reflect rarely used skills and were included to understand the scope of utilizing advanced physiologic monitors’ features. Thirty nurses from 4 adult ICUs were invited to respond to the survey.

**Results:**

Thirty nurses (100%) responded to the survey. The majority of nurses were from Neuro (47%) and Surgical Trauma (37%) ICUs. The data supported the high reliability and construct validity of the survey. At least one (3%) to 8 nurses (27%) reported lack of confidence on each item on the survey. On the first four subscales, 3% - 40% of the nurses reported they had never heard of or used 27 features/functions on the monitors. No relationships were found between subscales’ scores and demographic characteristics (*p* > .05). Nurses asked for training on navigating the central-station monitor and troubleshooting alarms, and the use of unit-specific super users to tailor training to users’ needs.

**Conclusion:**

This is the first study to create and test a list of competencies for physiologic monitors use. Rigorous, periodic and individualized training is essential for safe and appropriate use of physiologic monitors and to decrease alarm fatigue. Training should be comprehensive to include all necessary skills and should not assume proficiency on basic skills. Special attention should be focused on managing technical alarms. Increasing the number of super users is a recommended strategy for individualized and unit-specific training. There is a need for a usability testing of complex IT-equipped medical devices, such as physiologic monitors, for effective, efficient and safe navigation of the monitors.

## INTRODUCTION

Clinical alarm systems safety has received immense attention from clinicians, patient safety organizations, and policy makers [1-5], especially after the U.S. Food and Drug Administration’s (FDA) report indicated 566 alarm- related patient deaths [5]. The Joint Commission (TJC) in the U.S. attributed the majority of alarm-related deaths to alarm fatigue and subsequently issued a National Patient Safety Goal (NPSG) on clinical alarm systems safety [1]. TJC required healthcare organizations to implement the NPSG in two phases: Phase 1, beginning January 2014, was to identify high priority alarms, and Phase II, beginning January 2016, was to implement policies and procedures for alarm safety and to educate clinicians on alarm management [1]. In response to Phase II, this study assessed nurses’ perceived competence on physiologic monitors use in intensive care units (ICUs).

The FDA reported that physiologic monitors (also referred to as bedside or patient monitors), often used by nurses, were most commonly involved in alarm related-deaths [5]. The majority of studies on alarm systems safety verify the high rate of alarms produced by physiologic monitors, specifically in ICUs [6-14]. In fact, this rate reached 350 alarms per patient per day [3]. This high alarm rate can create alarm fatigue. Alarm fatigue occurs when clinicians ignore alarms from physiologic devices due to an excessive number of non-actionable or false positive alarms. Alarm fatigue can lead to unsafe workarounds, such as inappropriate disabling of alarms or unsafe adjustments to alarm limits, and was ranked by the ECRI Institute as one of the top 10 Technology Hazards [4].

Alarm systems safety is complex. Beyond the mere high rate of non-actionable alarms, safety can be compromised as alarms often look and sound alike [9, 10]. These similarities can decrease clinicians’ sensitivity to alarm urgency and affect clinicians’ responses to alarms. Moreover, such challenges raise questions about the usability of medical devices [9, 10]. Recent studies in ICUs show that alarm safety is affected by multifactors such as unit layout, lack of policies and procedures on alarm management, poor usability of physiologic monitors, lack of standardized practices on alarm reduction strategies, and most important to the current topic, lack of nurse competence on physiologic monitors use [9-13]. The need for system-based management strategies to improve alarm safety is well documented, especially in ICUs in which multiple monitoring devices are in constant use [1-4, 9-13]. On the other hand, although some system-based alarm management strategies achieved success in alarm reduction [14, 15], in other studies this success was not complemented with improvements in alarm fatigue or nurses’ attitudes toward alarm safety [9, 10]. Nurses reported the need for more training on physiologic monitors [9, 10]. It seems that the complexity of modern IT-equipped physiologic monitors requires structured individualized training focused on developing specific skill sets to navigate monitors and manage alarms [11, 12].

Studies on nurse training on alarm management are a few and tend to be focused on limited skills related to frequency and best practice in electrodes change and customizing parameters to be patient-specific [14, 15]. None of the previous studies outlined nurse competence on physiologic monitors use. To guide our initiatives and achieve Phase II of TJC’s clinical alarm systems safety NPSG, we created and validated a structured list of required alarm management skills and surveyed ICU nurses on their competence level on using physiologic monitors.

## MATERIALS & METHODS

### Design, Setting, and Sample

After obtaining approval of the Institute Review Board, this descriptive study took place in a Magnet hospital in a Southwestern state of the U.S. Nurses from 4 adult ICUs were invited to respond to a Nurse Competence on Physiologic Monitors Use Survey and were informed that the target sample size was 30 nurses because of budget constraints. Recruitment stopped after reaching a convenience sample of 30 nurses. ICUs were Medical (68 nurses), Transplant/Cardiac (49 nurses), Surgical Trauma (70 nurses), and Neuro ICU (43 nurses). New Philips physiologic monitors were deployed in all ICUs in April 2014. These included Philips IntelliVue MX800 bedside monitor, Philips IntelliVue Information Center iX central-station monitor, and Philips IntelliVue X2 transport monitor. Nurses received a structured group training of two hours demonstration on monitors use delivered by the company representative. If needed, individualized training was provided by their preceptors or unit educators.

### Instrument Development

The survey used in this study assessed level of perceived competence in using Philips physiologic monitors and consisted of 5 subscales with a total of 59 items. The subscales are admit, discharge, and transfer patient (5 items), hardware and connectivity (6 items), alarm management (19 items), appropriate monitoring (24 items), and advanced/specialized functions (5 items). Items on the first 4 subscales reflect most common tasks nurses from all adult ICUs perform using the monitors. Items on advanced/specialized functions subscale reflect rarely, if any, used skills and were included to understand the scope of utilizing advanced physiologic monitors’ functions/features. The survey was designed by the PI of the study, was guided by the user manuals of Philips cardiac monitors [16, 17], and was administered *via* SurveyMonkey. Manuals of cardiac monitors from other vendors (*i.e.*, General Electric) were also reviewed to increase the generalizability of the survey [18].

### Instrument Validation

The original survey included 63 items and went through validation by 13 expert ICU nurses, 3 from Medical ICU, 3 from Surgical Trauma ICU, 3 from Transplant/Cardiac ICU, and 4 from Neuro ICU. Nurse evaluators validated the appropriateness and relevancy of items and the fitness of items to the assigned subscales. Nurse evaluators were unit educators, with at least 5 years of ICU experience, super users of physiologic monitors, and members of the hospital-wide alarm systems safety taskforce.

Item relevancy and appropriateness were assessed using the following scale: (1) A commonly used skill and all nurses need to know about it, (2) A rarely used skill but nurses need to know how to do it, (3) A rarely used skill and nurses do NOT need to know about it or how to do it, and (4) Never heard of this function/feature. Items were considered relevant and included in the final survey if two conditions were met: (1) the item was rated as commonly used and needed or rarely used but needed by at least 75% of the evaluators and (2) the item was rated as rarely used and NOT needed by only one nurse evaluator. The latter condition was used to achieve a high agreement score (92%) on items that should be excluded from the competency list, therefore to focus attention on required skills. The latter condition excluded items under advanced/specialized functions subscale, as we anticipated that skills under this particular subscale were not commonly used or required for daily monitoring. Nevertheless, we wanted to gain more insight about bedside nurse use of and confidence in specialized features. Items under this subscale were mostly rated as not needed (by at least 2 nurse evaluators) or never heard of this feature (by at least 2 nurse evaluators). Only 5 nurse evaluators (38%) scored items under advanced/specialized functions subscale as commonly or rarely used but needed. All other items on the survey except four met the appropriateness and relevancy conditions and were included in the final survey. Excluded items were “Set date and time”, “Understand the meaning of the eleven types of arrhythmia beat labels”, “Adjust screen brightness”, and “Select the appropriate detection mode (*e.g.*, auto *vs.* manual respiration detection mode)”. Thus, after deleting these four items, the final survey had a total of 59 items. After validating item relevancy and appropriateness, the agreement between the 13 evaluators on the fitness of the 59 items to the 5 subscales was 100%.

The final survey included 3 sections. Section 1 collected information about demographic characteristics such as work unit, gender, age, work status, years of overall nursing experience, years of experience in the current unit, years of overall ICU experience, perceived level of computer expertise, whether nurses received training on physiologic monitors within the last 2 months, and whether they were super users for training on physiologic monitors.

Section 2 consisted of 59 scaled-items, scored on a 5-point Likert type scale, ranging from “1” for very unconfident to “5” for very confident, with an additional response option scored as “0” for never used or heard of this function/feature. In this section, nurses rated their skills when using bedside, central-station, and/or transport physiologic monitors. Items reflected knowledge, skills, and clinical reasoning/judgment in monitor use.

Section 3 included two open-ended items for nurses to (1) list other skills they think are relevant and essential for future in-service training, and (2) to indicate other tasks they frequently perform while using the physiologic monitors.

### Data Analyses

Means and proportions were used to describe nurse characteristics and their level of confidence at the item and subscale levels. Correlations between subscales and demographic variables and among subscales themselves were tested using Pearson’s *r* and χ^2^ analyses. Psychometric properties of the survey of internal consistency reliability and construct validity were assessed as part of the study.

## RESULTS

### Demographics

Thirty nurses responded to the survey assessing their level of perceived confidence in using Philips physiologic monitors. As seen in Table **1**, the majority of nurses were from Neuro (47%) and Surgical Trauma (37%) ICUs, female (83%), working full-time (60%), worked in their current unit for less than three years (63%), and had worked as a nurse for three or more years (57%). Most of the nurses indicated they had not received training on physiologic monitors within the last two months (83%), were not super users on physiologic monitors (83%), and rated their level of computer expertise as moderate or above moderate (97%).

### Perceived Level of Confidence

Items from the Nurse Competence on Physiologic Monitors Use Survey were evaluated based on 5 subscales. Table **2** presents the number of items in each subscale, subscale ranges, means, and standard deviations, and Cronbach's Alpha internal consistency coefficients. Nurses’ level of confidence was highest in the domain of appropriate monitoring (77^th^ percentile), somewhat lower in the domains of admit, transfer, and discharge patients (74^th^ percentile) and alarm management (73^rd^ percentile), then by hardware and connectivity (70^th^ percentile), and lowest in the domain of advanced functions (39^th^ percentile).

Preliminary evaluation of reliability was adequate in all subscales (*i.e.*, Cronbach’s alpha ranging from 0.71 to 0.91). To examine the strength of relationships across the five survey subscales, Pearson’s correlation coefficients were generated. Interpretation of findings indicated all subscales were significantly and positively related to one another (*r* ranging from 0.59 to 0.84, P > .0001), providing support for the construct validity of the survey.

When examined for missing responses, 5 of the 59 items (8.5%) had one or two missing responses. Items were categorized into 4 main categories based on mean scores. A mean of 0 score indicates “Never used/heard of this function”. Items with mean scores from 1.00 - 2.99 indicate nurses were “Not confident”. Items with mean scores from 3.00 to 3.99 were categorized as “Neutral”. Items with mean scores from 4.00 to 5.00 indicate nurses were “Confident”.

As seen in Table **3**, the range of reported confidence on the subscales’ items was 33% - 87% of the nurses on admit, discharge and transfer patient subscale, 17% - 83% on hardware and connectivity, 17% - 93% on alarm management, 33% - 97% on appropriate monitoring, and 3% to 50% on advanced/specialized functions. At least one (3%) to 8 nurses (27%) reported lack of confidence on each item on the survey. Of the nurses, 20% to 27% reported lack of confidence on the following items: transfer patient from central and bedside monitors (23%, Item 3), edit patient information after admission (23%, Item 4), resolve patient information mismatch (23%, Item 5), report device malfunctions to service personnel (20%, Item 8), customize default settings to patient specific (20%, Item 24), troubleshoot common technical alarm messages (23%, Item 25), eliminate redundant alarms when changing default settings (27%, Item 26), know when to contact service personnel to correct technical alarms (23%, Item 28), extend alarm pause time (23%, Item 29), adjust speed of waves (20%, Item 45), freeze and unfreeze waves (20%, Item 47), recognize when specific monitoring is needed (20%, Item 48), differentiate/print patient reports (23%, Item 53), and disable/re-enable monitor touchscreen operation (23%, Item 54). On the first four subscales, 3% - 40% of the nurses reported they had never heard of or used 27 features/functions on the monitors and 20% to 63% reported they had never heard of or used all features on the advanced/specialized functions subscale.

A series of comparison tests of χ^2^ were performed to examine if subscales’ scores differed as a function of demographic characteristics (*i.e.*, age, ICU type, work status, years of experience as a nurse, years in current unit, and years in ICU). No differences were noted across analyses (*p* > .05).

### Narrative Data

A total of 6 nurses (20%) listed other skills they felt were relevant and essential for future in-service training. These skills were understanding how bedside monitors transmit data to the main terminal (central-station monitor) and the limitations exist between the two monitors (Neuro and Transplant ICU nurses), navigating and function review of the central-station monitor (Neuro ICU nurse), and optimizing the use of the monitors and troubleshooting when the bedside monitor does not recognize the X2 transport monitor (Surgical Trauma ICU nurse). A Neuro ICU nurse mentioned that each ICU is specific in their use of the monitors and that having unit-specific super users would be helpful in tailoring training to user needs. A Surgical Trauma ICU nurse noted the need to enlarge the tele-monitoring window in the bedside monitor due to its small size. This issue arises when a nurse is simultaneously tele-monitoring a patient from the bedside monitor of another patient. In such cases, the tele-monitored patient data is displayed in a subscreen that occupies approximately 25% of the total monitor screen.

A total of 5 nurses listed other tasks they perform frequently when using the monitors. Most tasks were related to monitoring unit-specific parameters, such as pulmonary artery wedge pressure measurements, cardiac output thermodilution measurements, pulmonary artery pressure continuous readings (Transplant ICU nurses), central venous pressure, corrected QT interval, and monitoring intracranial pressure (ICP) using two ICP lines at the same time (Neuro ICU nurses).

## DISCUSSION

In this study, we created and validated a set of competencies and surveyed bedside ICU nurses on their knowledge and perceived confidence in using physiologic monitors. Our initial intent was to gain information that would guide future in-service training on physiologic monitors use. Ultimately, we hope to develop a standardized self-assessment checklist that adult ICU nurses can use to evaluate and enhance alarm systems management and safety practices.

Excluding the 5 items under advanced/specialized functions subscale, 1 to 7 nurses (3% - 23%) reported a lack of skills or knowledge to perform one or more of the remaining 54 functions/features of the physiologic monitors. All of these items were rated as needed by the expert nurses who validated the survey, of particular interest, at least one fifth of the nurses reported a lack of confidence in 17 essential functions. Some of these functions were related to alarm management and appropriate monitoring, such as extending alarm pause time. Additionally, 9 (30%) nurses reported not using or had never heard of this particular function, indicating the need for future in-service. Extending alarm pause time is an important alarm management strategy to decrease the number of false alarms and alarm fatigue, especially for care procedures that last longer than 1 minute, which is the default setting of alarm pause in our system that can be extended to 5 or 10 minutes. On the other hand, some institutions may discourage extending the pause period for safety purposes.

There was also a reported lack of confidence in customizing default settings to be patient specific, recognizing when specific monitoring is needed for specific medical cases, and in eliminating redundant alarms when changing default settings. These skills are essential for safe monitoring and to decrease alarm fatigue and the overall number of alarms.

Our data also support the need to improve nurses’ skills on troubleshooting technical alarm messages and monitoring problems, when to contact service personnel and how to report device malfunctions. More than 20% of the nurses reported lack of confidence in these skills and up to 3 (10%) reported they had never used or heard of these functions. The narrative data also indicated the need for future in-service training on these skills. The absence of a standardized reporting system for device malfunctions is likely to contribute to this problem.

There are many types of Technical alarms. Examples of such alarms include “Empty Battery”, an alarm triggered because the remaining charge/power in the battery to operate the device is down to 10 minutes. This alarm cannot be paused or silenced and should be acknowledged by replacing the battery or connecting the device to the charger. To troubleshoot this particular alarm, nurses need to know how to identify the battery's power status of the X2 monitor from the display color, which is one of the front panel color indicators or LEDs (Item 11). Up to 11 (37%) nurses reported they had never heard of these functions. Other technical alarms include “ECG Leads Off”, which is triggered when leads are displaced or not all required leads are attached, and “Check Patient ID”, an alarm triggered when there is a mismatch in patient data between two monitoring devices connected to the patient. Nurses’ lack of competence in troubleshooting “Check Patient ID” technical alarm may explain nurses’ limited confidence in editing patient information after admission as well as resolving patient information mismatch (Items 4 and 5).

Troubleshooting monitoring problems is essential for alarm management. Most of the research on alarm management focuses attention on physiologic alarms [6-8]. Little information is available on the rate of technical alarms, which also contribute to alarm fatigue [19]. In a previous study [10], Sowan and colleagues analyzed data logged from physiologic monitors in a Transplant Cardiac ICU for a 10-week period and found a total of 9224 technical alarms (unpublished results, personal communication with the authors), equivalent to a rate of 12.6 alarm/patient day, with the most common being ECG Leads Off (a total of 5552 alarms).

Lack of confidence in different skills may be related to lack of use (or rarely used functions), lack of training on physiologic monitors, and/or lack of usability of physiologic monitors. However, skills such as admit and discharge a patient, connect monitor cables, place electrodes, identify major hardware components, pause and silence alarms, know parameters’ display and the meaning of waves, view active alarm messages and differentiate priority, change alarm limits safely, easily and appropriately, and many other skills are all commonly used in any adult ICUs on daily basis and essential for safe and appropriate monitoring. Having nurses reported lack of confidence in these fundamental skills strongly supports the need for training and examining the usability of physiologic monitors. Our results suggest poor usability of some features of the monitors. For example, 4 to 5 (13% - 17%) nurses reported lack of confidence in navigating different monitors' screens easily and efficiently (Items 43 and 52). Other nurses reported they had never heard of or used these functions. Furthermore the lack of confidence of the nurses in identifying the difference between latching and non-latching alarms and the high reported percentage (40%) on never heard of this function can be related to the look-alike and sound-alike alarms [10], another usability-related issue. Since ease-of-use is a fundamental usability attribute, these findings, and similar to previous findings [9, 10, 20], further support the need to examine the usability of physiologic monitors.

In the narrative data, nurses asked to focus the in-service on functions review and appropriate navigation of the central-station monitor. They also wanted to understand the data transmission process from the bedside to the central monitor, and the limitations of the two monitors. Although many of the functions/skills included in the survey are applicable to bedside, central-station and transport monitors, the processes of completing tasks and screen navigation differ based on the monitor in use. For example, the steps to admit a patient are different when using the central-station versus the bedside monitor, requiring nurses to master multiple skill sets. Additionally, some skills are limited to specific monitors, such as clearing sectors, unit management of log alarm data, and locating equipment, which are limited to the central-station monitor. Our survey did not focus on the specific skills limited to the central-station monitor.

Although skills on the survey were applicable to all adult ICUs and some items were written in general terms such as “know different types of parameters display”, patients’ medical conditions require knowledge of case-specific parameters. Therefore, the suggestion to have unit-specific super users is vital. For example, Neuro ICU nurses reported a concurrent monitoring of 2 ICP readings, a parameter that may not be frequently monitored in other ICUs. Monitoring 2 ICP measurements requires nurses to pick an alternative waveform for the second ICP measurement. In this case, it is important to know the capabilities of the physiologic monitor in terms of the maximum number of waveforms that can be displayed. Our bedside monitors allow simultaneous display of 8 waveforms. So, for example, if nurses are monitoring the waves of Lead 1, Lead 2, Pulse, NBP (noninvasive blood pressure), CVP (central venous pressure), ABP (arterial blood pressure), Respiration, and one ICP, they need to eliminate one of these measurements to monitor a second ICP waveform. Nurses usually sacrifice the Respiration waveform. The nurse decision on the parameter to sacrifice should be carefully made based on the client condition and the possibilities for risking safety. This issue, for example, can be one of the critical unit-specific training aspects.

In regard to advanced functions, more than one third of the nurses were either not aware or not using the following features: sepsis protocol/guidelines, manual entry of data into the monitor, and drug calculator. Not using sepsis protocol can be related to the fact that a sepsis protocol is embedded into the electronic medical record (EMR), reducing the need for such feature within the physiologic monitors. On the other hand, this feature can be useful for hospitals with no e-protocol for sepsis and when complete interoperability exists between the EMR and physiologic monitors. Similarly, manual entry of lab data into the monitor may not be usable, specifically when there is no algorithm to connect lab data and the monitored parameters to help in clinical decision-making. Likewise, the use of drug calculator within the monitor can be inconvenient for nurses, given the availability and ease of access to pocket calculators, cell phones, and/or drug calculation apps. When hospitals select their physiologic monitors and medical devices, a thorough discussion with the vendor is recommended to ensure optimal use of advanced features of their products. Unfortunately, little research is available about the use of such features within the physiologic monitors.

Our survey includes skills required by all bedside ICU nurses based on our institution’s policies and procedures of safe monitoring. In other hospitals, some of these skills may be performed by nurse practitioners, super users, certified critical care nurses, or physicians. Therefore, adhering to unit policies and procedures is important when surveying nurses about their competence on physiologic monitors use. Additionally, a system for periodic assessment, training, and documentation of nurse competence on physiologic monitors use should be in place for safe monitoring.

In previous projects on alarm systems safety, assembling a multidisciplinary alarm management team, changing alarm default settings, having a unit policy on frequency of electrodes’ replacement, and providing standardized in-service nursing training on physiologic monitors use were insufficient to reduce alarm fatigue, despite a significant reduction of 25% in alarm rate [10]. Lack of competence on physiologic monitor use is a root cause of alarm fatigue [9, 10]. The educational aspect of this complex problem can be improved by focusing attention on the skills nurses need to effectively, efficiently and safely navigate physiologic monitors. Our competence list includes basic navigation skills for all adult ICUs. Detailing unit-specific skills can expand the list and super users should facilitate the competency process.

The findings of this study should be interpreted in light of the following limitations. Although we had a motivated sample, our sample size was small due to budget constraints. Including a larger sample of nurses would improve our knowledge about the essential skills in physiologic monitors use in adult ICUs and improve the generalizability of the study and survey. Extending the survey to pediatric and neonate ICUs may also be beneficial to understand unit-specific skills in monitoring pediatric and neonate patients. Although our survey was designed based on the features of Philips and General Electric Monitors, many items on the survey are applicable to all physiologic monitoring devices irrespective of the manufacturer. The main difference among physiologic monitors from different vendors can be related to the availability of advanced features (*e.g.*, sepsis protocols). On the other hand, examining the survey against the functions of other monitoring devices may increase its generalizability.

## CONCLUSION

This is the first study to create and test a list of competencies for physiologic monitor use. Rigorous, periodic and individualized training is essential for safe and appropriate use of physiologic monitors and to decrease alarm fatigue. Training should be comprehensive to include all necessary skills and should not assume proficiency on basic skills, such as admitting a patient to the monitor. Special attention should be focused on managing technical alarms. Increasing each unit’s number of super users is a recommended strategy for individualized and unit-specific training. There is a need for usability testing of complex IT-equipped medical devices such as physiologic monitors for effective, efficient and safe navigation.

## Figures and Tables

**Table 1:** Demographic characteristics of nurse respondents (N= 30).

**Characteristic**	**n (%)**		**Characteristic**	**n (%)**
ICU			Years working in intensive care units	
Neuro	14 (47%)		Less than 3 years	15 (50%)
Surgical trauma	11 (37%)		3 or more years	15 (50%)
Medical	3 (10%)			
Transplant cardiac	2 (7%)		Years working as a nurse	
			Less than 3 years	13 (43%)
Age group			3 or more years	17 (57%)
Less than 30 years	14 (47%)			
30 or more years	16 (53%)		Trained within last 2 months^a^	
			No	25 (83%)
Gender			Yes	3 (10%)
Female	25 (83%)			
Male	5 (17%)		Serve as training super user	
			No	25 (83%)
Work status			Yes	5 (17%)
Full-time	18 (60%)			
Part-time	12 (40%)		Level of computer expertise	
			Novice	1 (3%)
Years working in unit			Moderate	18 (60%)
Less than 3 years	19 (63%)		Above moderate	11 (37%)
3 or more years	11 (37%)		Expert	0

^a^ Percentage does not equal 100 due to missing responses.

**Table 2:** Survey subscales, number of subscale items, and reliability coefficients.

**Subscale title**	**No. of items (Range)**	**Mean (SD** ^a^ **)**	**Cronbach's alpha**
Appropriate monitoring	24 (0 - 120)	92.30 (18.09)	0.91
Admit, discharge, transfer patient	5 (0 - 25)	18.50 (4.83)	0.84
Alarm management	19 (0 - 95)	69.00 (16.22)	0.93
Hardware and connectivity	6 (0 - 50)	20.97 (5.72)	0.72
Advanced/Specialized functions	5 (0 - 25)	9.76 (5.54)	0.71

^a^ SD is the standard deviation.

**Table 3:** Percent of nurse responses to competence on physiologic monitors use items organized by subscales and confidence level (N=30).

**Item No.**	**Assessment items by subscale**	**Percent of nurses (%)**			
		**Confident**	**Neutral**	**Not confident**	**Never used**
**1. Admit, Discharge, and Transfer Patient**					
1	Discharge patient from central and beside monitors	87	0	13	0
2	Admit patient to central and bedside monitors	87	3	10	0
3	Transfer patient from central and bedside monitors	53	20	23	4
4	Edit patient information after admission	50	20	23	7
5	Resolve patient information mismatch (*e.g.*, between X2^a^ and bedside monitor, or bedside and central monitors)	33	44	23	0
**2. Hardware and Connectivity**					
6	Connect monitor cables	83	0	17	0
7	Identify monitors’ major hardware components and connectors (SpO2^b^, NBP^c^, *etc.*)	80	3	17	0
8	Report device malfunctions to service personnel	70	3	20	7
9	Identify battery's power status of X2^a^ monitor from display color (green, yellow, or red)	57	13	17	13
10	Clean, sterilize and disinfect monitors and monitors accessories	57	20	17	6
11	Describe the functions of alarm lamps and front panel color indicators (or LEDs^d^)	17	36	10	37
**3. Alarm Management**					
12	Pause alarms and cancel the pause	93	0	7	0
13	Silence alarms	83	0	10	7
14	Know different types of parameters’ display and the meaning of waves and information in the display (*e.g.*, arrhythmia, SpO2^b^, Respiration, *etc.*)	80	10	10	0
15	View all active alarm messages easily	80	3	17	0
16	Change alarm volume easily	80	3	7	10
17	Choose and change the source (*e.g.*, Systolic, Mean, Systolic and Mean) of an alarm appropriately (*e.g.*, pressure alarms source, NBP ^c^, *etc.*)	77	13	10	0
18	Change alarm limits safely and appropriately	73	10	17	0
19	Change alarm limits easily	73	10	17	0
20	Identify and differentiate the priority (*e.g.*, from crisis to advisory) and meaning of all physiologic alarm messages, based on visual and audible alarm indicators	73	10	17	0
21	Acknowledge and correct alarm messages appropriately	73	17	10	0
22	Identify and differentiate the priority and meaning of technical alarm messages (*e.g.*, Check Equipment), based on visual and audible alarm indicators	70	13	17	0
23	Differentiate the source of each alarm (*e.g.*, HR^e^ Low alarm is from ECG^f^ settings)	70	20	10	0
24	Customize default settings to patient specific	67	13	20	0
25	Troubleshoot common technical alarm messages (*e.g.*, !!Check Patient ID)	57	17	23	3
26	Eliminate redundant alarms when changing default settings (*e.g.*, if ST^g^ and STE^h^ are selected, STE^h^ will be redundant alarms)	53	17	27	3
27	Understand the monitor logic behind displaying different types of alarms (*e.g.*, if there is an active ventricular bigeminy alarm, a PVC^i^ > 6/min will NOT be triggered because it is lower in the same priority chain)	53	23	17	7
28	Know when you need to contact service personnel to correct technical alarms *vs.* when you need to troubleshoot the problem	50	17	23	10
29	Extend alarm pause time	30	17	23	30
30	Differentiate the behaviors of latching (alarm automatically will turn off when the condition no longer exists) *vs.* non-latching alarms (require nurse to turn alarm off even if the condition no longer exists)	17	33	10	40
**4. Appropriate Monitoring**					
31	Place electrodes appropriately	97	0	3	0
32	Understand best practices in electrode placement (frequency of changing electrodes, skin preparation)	97	0	3	0
33	Change the NBP^c^ measurement interval	97	0	3	0
34	Select the appropriate NBP^c^ measurement modes (manual, auto, sequence, stat)	90	3	7	0
35	Store and send the 12-lead ECG^f^ to the central monitor	90	3	7	0
36	Zero the pressure transducer	90	0	7	3
37	Put monitor into Standby mode and resume from Standby monitoring	90	7	3	0
38	Select appropriate invasive pressure label for monitoring (*e.g.*, ABP^j^, ICP^k^, PAP^l^, Ao^m^)	87	6	7	0
39	Change the size of a waveform	83	0	17	0
40	Select optimal SpO2^b^ measurement site	83	4	13	0
41	Recognize elements and purpose of using monitors’ Screen Keys: (1) The four permanent keys (Silence, Pause Alarms, Main Setup, Main Screen), (2) smart keys, and (3) pop-up keys	80	13	7	0
42	Pick best primary and secondary leads for paced and non-paced patients	77	3	17	3
43	Navigate the different monitors' screens easily	77	6	17	0
44	Check beats annotation and relearn arrhythmia analysis	73	10	10	7
45	Adjust speed of different kinds of waves	67	6	20	7
56	Pick the appropriate lead for ST^g^ monitoring	67	16	7	10
47	Freeze and unfreeze waves	63	10	20	7
48	Recognize when specific monitoring is needed for specific patient cases (*e.g.*, ST^g^-Segment monitoring, QT^n^ monitoring)	63	14	20	3
49	Recognize patient cases when specific monitoring is NOT recommended or clinically insignificant (*e.g.*, in cases of setting ST^g^ or QT^n^ monitoring)	63	21	13	3
50	Review trended patient data using screen trends	60	16	7	17
51	Explain the information displayed in trend windows	60	10	10	20
52	Use shortcuts to navigate monitor screens and keys efficiently (*e.g.*, Select Quick Admit Smart Key to quickly admit a patient)	43	27	13	17
53	Differentiate/print patient reports available within the monitor	40	20	23	17
54	Temporarily disable/re-enable monitor touchscreen operation	33	24	23	20
**5. Advanced Functions**					
55	View hemodynamic, oxygenation, and ventilation calculations	50	13	17	20
56	Perform parameters calculations	37	26	10	27
57	Access/use the drug calculator from the monitor	20	20	17	43
58	Manually enter some data into the monitor (*e.g.*, lab results)	7	29	17	47
59	Use sepsis protocol and its guidelines that are within the monitor	3	21	13	63

^a^X2: is the transport monitor. ^b^SpO2: peripheral capillary oxygen saturation. ^c^NBP: noninvasive blood pressure. ^d^*LED:* light-emitting diode. ^e^HR: heart rate. ^f^ECG: electrocardiogram. ^g^ST: ST segment in the electrocardiogram. ^h^STE: ST elevation. ^i^PVC: premature ventricular contraction. ^j^ABP: arterial blood pressure. ^k^ICP: intracranial pressure. ^l^PAP: pulmonary artery pressure. ^m^Ao: aortic pressure. ^n^QT: QT segment in the electrocardiogram.

## LIST OF ABBREVIATIONS

(FDA): = Food and Drug Administration
(TJC): = The Joint Commission
(NPSG): = National Patient Safety Goal
(ICUs): = Intensive care units
(ECG): = Electrocardiogram
(ICP): = Intra cranial pressure
(NBP): = Noninvasive blood pressure
(CVP): = Central venous pressure
(ABP): = Arterial blood pressure
(EMR): = Electronic medical record
